# Supplementary material for: Causal Relationship between Aging and Anorexia Nervosa: A White-Matter-Microstructure-Mediated Mendelian Randomization Analysis
Source: Biomedicines. 2024 Aug 16;12(8):1874. doi: 10.3390/biomedicines12081874 (PMC11351342; doi:10.3390/biomedicines12081874)
Supplement: Supplementary file 1 [file biomedicines-12-01874-s001.zip › Supplementary Figure/Figure S4/Singe SNP plot of MR analysis for TL to ubm-b-1863.pdf]

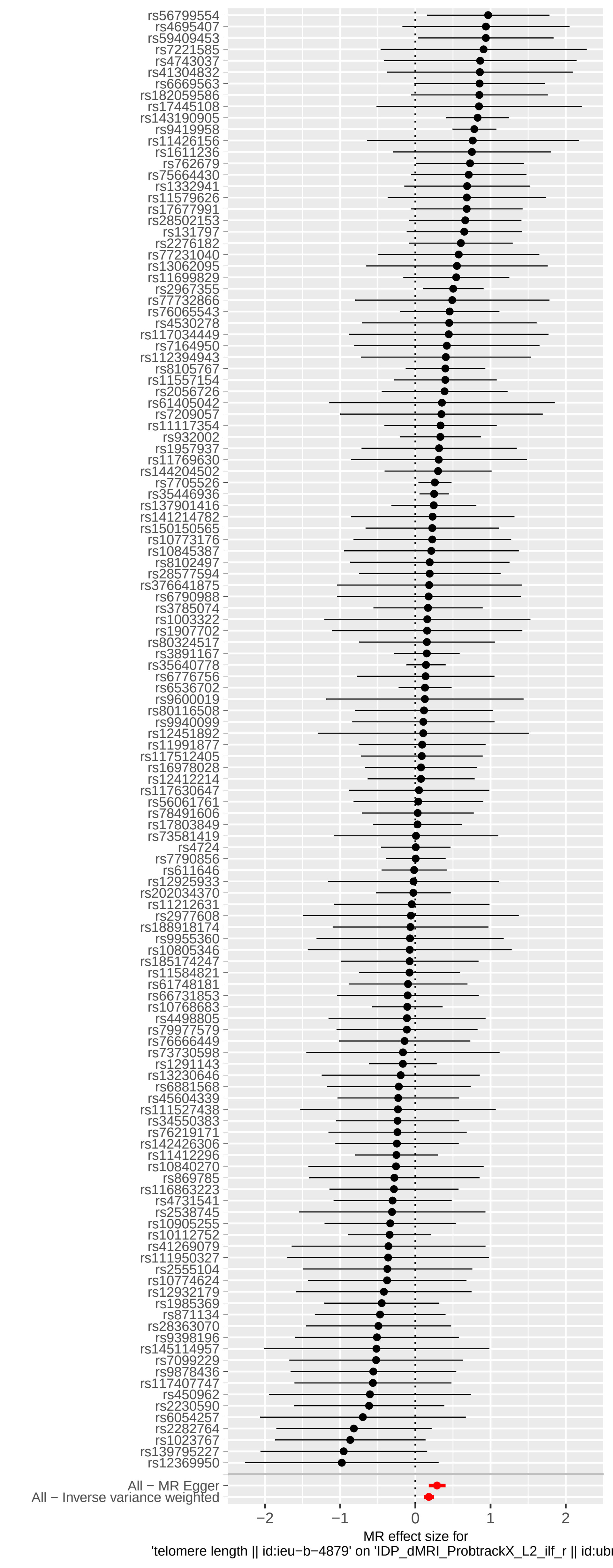

rs56799554
rs4695407
rs59409453
rs7221585
rs4743037
rs41304832
rs6669563
rs182059586
rs17445108
rs143190905
rs9419958
rs11426156
rs1611236
rs762679
rs75664430
rs1332941
rs11579626
rs17677991
rs28502153
rs131797
rs2276182
rs77231040
rs13062095
rs11699829
rs2967355
rs77732866
rs76065543
rs4530278
rs117034449
rs7164950
rs112394943
rs8105767
rs11557154
rs2056726
rs61405042
rs7209057
rs11117354
rs932002
rs1957937
rs11769630
rs144204502
rs7705526
rs35446936
rs137901416
rs141214782
rs150150565
rs10773176
rs10845387
rs8102497
rs28577594
rs376641875
rs6790988
rs3785074
rs1003322
rs1907702
rs80324517
rs3891167
rs35640778
rs6776756
rs6536702
rs9600019
rs80116508
rs9940099
rs12451892
rs11991877
rs117512405
rs16978028
rs12412214
rs117630647
rs56061761
rs78491606
rs17803849
rs73581419
rs4724
rs7790856
rs611646
rs12925933
rs202034370
rs11212631
rs2977608
rs188918174
rs9955360
rs10805346
rs185174247
rs11584821
rs61748181
rs66731853
rs10768683
rs4498805
rs79977579
rs76666449
rs73730598
rs1291143
rs13230646
rs6881568
rs45604339
rs111527438
rs34550383
rs76219171
rs142426306
rs11412296
rs10840270
rs869785
rs116863223
rs4731541
rs2538745
rs10905255
rs10112752
rs41269079
rs111950327
rs2555104
rs10774624
rs12932179
rs1985369
rs871134
rs28363070
rs9398196
rs145114957
rs7099229
rs9878436
rs117407747
rs450962
rs2230590
rs6054257
rs2282764
rs1023767
rs139795227
rs12369950
All − MR Egger
All − Inverse variance weighted
−2
−1
0
1
2
MR effect size for
'telomere length || id:ieu−b−4879' on 'IDP_dMRI_ProbtrackX_L2_ilf_r || id:ubm
